# Health Equity Impact Assessment Related to Air Pollution Reduction

**DOI:** 10.3390/ijerph192215352

**Published:** 2022-11-21

**Authors:** Séverine Deguen, Wahida Kihal-Talantikite

**Affiliations:** 1 PHARes Population Health trAnslational Research Inserm CIC 1401, Bordeaux Population Health Research Center, Bordeaux University, 33000 Bordeaux, France; 2 LIVE UMR 7362 CNRS (Laboratoire Image Ville Environnement), University of Strasbourg, 67000 Strasbourg, France

Despite considerable improvements in terms of prevention, management, and regulation, air pollution remains a leading environmental health issue worldwide. In 2019, the World Health Organization (WHO) estimated that 99 out of 100 people breathe polluted air and live in places where air quality levels exceed the limits set out by the WHO [1]. While the health risk of air pollution is relatively low compared to other risk factors (such as alcohol or tobacco consumption, for instance), the total number of affected people is significant. A recent Lancet Commission report states that, in 2019, about 4.5 million deaths were associated with ambient air pollution exposure. The report also mentioned the fact that the number of premature deaths is rising annually, from 2.9 million in 2000 to 4.2 million by 2015 [2].

However, most studies have either focused on estimating the relationship between pollution and health or the health benefits arising from reduced air pollution. Less attention has been paid to the differential health consequences of air pollution exposure according to socioeconomic status, whether measured at individual and/or neighborhood levels, or over a different lifespan. Yet, it is now well established that social inequalities combined with environmental exposure can increase health risks through two main mechanisms. Firstly, the exposure differential means that the most deprived populations (or people living in deprived areas) are exposed to a higher number of environmental nuisances. Secondly, the vulnerability differential refers to the increasing sensitivity of the most deprived populations when exposed to environmental nuisances (including ambient air pollution exposure). More specifically, it means that socioeconomic status can modify the health consequences of air pollution exposure as a result of either poorer living conditions or reduced access to the healthcare system. In Paris in 2015, Deguen et al. demonstrated a strong association between short-term variations in NO_2_ concentrations (measured at census block scale) and all-cause mortality for adults living in low-socioeconomic-status areas [3].

Alongside this, national plans covering multiple domains (nutrition, environment, cancer, etc.) that aim to improve the population’s health fail to consider the health risk differential relating to individual socioeconomic status or the socioeconomics of residential areas. Specifically, the translation of national plans into actions does not integrate differences in implementation according to social disadvantages that could lead to increased social health inequalities. This means that even when an intervention reducing air pollution does lead to health benefits, these benefits are not equally distributed across the population in terms of socio-economic status. Health inequalities are also linked to the organization, management and the use of space (whether urban, peri-urban or rural), marked by a segmentation that is directly linked to land value and the price of houses. Due to the absence of deliberate social mixing policies, socially segregated blocks are created. These gradually shift towards social invisibility because of social groups’ unequal ability to articulate and assert their interests.

Quantitative Health Impact Assessments (HIAs) stand out as one of the best decision-making policy guidance tools for the design of interventions that support environmental policies aiming to tackle air pollution. HIAs can provide useful and valuable information regarding the future health effects of a potential plan or policy. Several worldwide interventions that aim to reduce air pollution have been implemented and quantitatively evaluated for health impacts. In a recent systematic review of the literature, Burns et al. [4] identified 42 studies (published prior to 2016) evaluating interventions that aim to reduce air pollution; of these, 38 interventions were evaluated. The majority of interventions focused on traffic (n = 22), and intervention assessments and were most often based on environmental indicators of air pollution. The second type of impact studied was health, and, more rarely, other impacts (such as economic impacts) were considered. Of the 42 studies, 11 were conducted in urban environments in European countries. These interventions were carried out in Germany, Spain, Ireland, Italy, the Netherlands, the United Kingdom, and Slovenia. Most European interventions concerned road traffic (n = 10): the implementation of low-emission zones (LEZ), urban tolls, the reduction in speed limits, increase in public transport options (or modification of existing lines), and the opening of a bypass road to decrease road traffic in the urban center. Interventions assessments focus on air pollution indicators, comparing levels before/after the intervention and/or with ‘control’ areas. The most commonly used air pollution indicators were nitrogen oxides (NO_x_), including NO_2_, and fine particles (PM10 and PM2.5). Less frequently, other pollutants were used (ozone, black smoke, soot carbon, SO_2_). Only two studies evaluated health interventions as well as environmental impacts [5,6]. The health indicators used were the change in respiratory symptoms (collected via a respiratory health survey in the intervention and control areas) [6] and mortality and hospitalizations for cardiovascular, respiratory or digestive causes [5]. The two studies evaluating the health effects of the intervention found improvements based on several health indicators, such as the reduction in cases of rhinitis and rhino conjunctivitis symptoms following the opening of a city bypass road in the UK, and a 17% reduction in respiratory mortality associated with the ban on coal in Dublin.

In addition, without being based on any actual intervention or action, many other studies have estimated the reduction in premature mortality resulting from a hypothetical reduction in air pollution: a decrease of 1–2% in total annual all-cause mortality in the urban area of Lausanne-Morges in Switzerland [7] and a decrease of 3–8% in Grenoble and Lyon in France, attributable to PM2.5 exposure [8]. To our knowledge, however, few published epidemiological studies have examined the impact on health equity of either a hypothetical reduction in air pollution or an anticipated reduction following an intervention.

This Special Issue aims to summarize the current state of affairs in studies quantifying the health benefit of air pollution reduction by population subgroups, according to their socio-economic status. This constitutes the preliminary step that is necessary for the production of a robust dose–response function in socio-economic groups, followed by estimation of health benefits by group.
